# Modeling the Distribution of Medically Important Tick Species in Florida

**DOI:** 10.3390/insects10070190

**Published:** 2019-06-28

**Authors:** William H. Kessler, Claudia Ganser, Gregory E. Glass

**Affiliations:** 1 Department of Geography, University of Florida, Gainesville, FL 32611, USA; 2 Department of Geography, Emerging Pathogens Institute, University of Florida, Gainesville, FL 32611, USA

**Keywords:** Ixodid ticks, distribution, geography, modeling, ensemble, niche, Lone star, Black-legged, American dog

## Abstract

The lone star (*Amblyomma americanum*), black-legged (*Ixodes scapularis*) and American dog ticks (*Dermacentor variabilis*) are species of great public health importance as they are competent vectors of several notable pathogens. While the regional distributions of these species are well characterized, more localized distribution estimates are sparse. We used records of field collected ticks and an ensemble modeling approach to predict habitat suitability for each of these species in Florida. Environmental variables capturing climatic extremes were common contributors to habitat suitability. Most frequently, annual precipitation (Bio12), mean temperature of the driest quarter (Bio9), minimum temperature of the coldest month (Bio6), and mean Normalized Difference Vegetation Index (NDVI) were included in the final models for each species. Agreement between the modeling algorithms used in this study was high and indicated the distribution of suitable habitat for all three species was reduced at lower latitudes. These findings are important for raising awareness of the potential for tick-borne pathogens in Florida.

## 1. Introduction

In Florida, cases of locally acquired tick-borne illnesses are reported yearly [1]. Officially recognized, locally acquired cases are sporadic, which may reflect variation in the types of human activities that influence exposure or transmission risk and/or variation in the distribution of vector species. Distribution estimates are often restricted to county level averages when detailed spatial information is unavailable or when the focus is on large geographic extents. While this approach can often leverage available data, it can mask important nuances in the distribution of exposure risk. In this paper, we address the latter aspect of vector-borne disease risk and this necessitates examining vector distributions at resolutions fine enough to associate human behaviors with the local environments. This requires discerning patterns at a spatial resolution representing the sub-county scales.

One of the major methodological challenges in characterizing tick vector distributions is that there are many statistical and pattern matching approaches to estimate the distribution of the vector [2]. All these approaches rely on identifying suitable habitat for a species based on associations between observed presence/abundance and environmental characteristics of those locations [3]. However, each modeling approach uses method-specific parameters and assumptions that may entrain unrecognized predictive biases unrelated to the biology of the species being modeled [4]. One solution to reduce the unrecognized biases is to use ensembles of different modeling algorithms with the presumption that their overall patterns will reduce the potential predictive bias produced by any single model [5,6]. Ensemble outputs highlight areas of agreement across predictions and are believed to indicate greater confidence in the suitability of those locations for the target (tick) species [5].

Many previous efforts of ensemble models for tick species’ distributions have been done at regional scales and relied on limited sets of environmental (primarily climatic) data [7,8,9,10,11]. Here, we use an ensemble prediction from five common modeling algorithms to estimate the geographic distributions of suitable habitat for the lone star (*Amblyomma americanum*), black-legged (*Ixodes scapularis*), and American dog ticks (*Dermacentor variabilis*), three species of public health importance in Florida. These models are developed at a resolution of one hectare, to provide a more detailed scale for case investigation, or intervention.

Nationally, the incidence of tick-borne diseases is increasing and represents an overwhelming majority of vector-borne infections in the U.S. each year [12]. Reports of these diseases have outpaced other vector-borne illness by a factor of 9:1 between 2004 to 2013 [12]. Lone star, American dog, and black-legged ticks are known to transmit several of the most frequently reported pathogens causing disease in humans. The black-legged tick is the primary vector for Lyme disease pathogens (*Borrelia* spp.) in the Eastern United States, as well as a vector for *Anaplasma phagocytophilum*, Ehrlichiosis causing agents, and the parasite *Babesia microti* [13,14,15,16,17]. Cases of Lyme disease account for nearly 70% of all nationally notifiable vector-borne diseases in the U.S. [12]. High abundance, voracious and nonspecific biting habits, and known or suspected competence for transmission of *Ehrlichia chaffeensis* and *E. ewingii*, *Francisella tularensis*, and the agent of southern tick-associated rash illness (STARI) make the lone star tick a species of significant public health concern [18,19,20,21,22,23,24,25,26,27]. The American dog tick is an important vector of both human and animal pathogens. This species is known to transmit *Rickettsia rickettsii* (Rocky Mountain spotted fever—RMSF), *Francisella tularensis*, and other notable pathogens [28,29].

In this study we used five modeling algorithms: Generalized linear models (logistic regression), boosted regression trees, random forests, multivariate adaptive regression splines, and maximum entropy to estimate the distribution of lone star, black-legged, and American dog ticks. The five modeling algorithms used in this study have been used in various ecological applications to estimate the geographic distributions of numerous vagile and non-vagile species, including ticks [11,30,31]. Common species distribution modeling (SDM) algorithms generally fall into one of two categories: Statistical approaches, which rely on underlying assumptions about the distributions of data values or models; and machine learning approaches, which instead group the data into distinct, homogeneous groupings (classification) without assuming an a priori distribution [32].

Traditional statistical approaches stem from regression methods and include generalized linear models (GLMs), and their extensions, such as generalized additive models (GAMs) and multivariate adaptive regression splines (MARS). Logistic regression (GLM with a logit link function) is a common statistical method to model binary responses such as presence/absence data. The method is well established and easily interpretable. The response variable from logistic regression is a probability that the outcome is the value of interest that is conditional on the weighted linear combination of predictor variables [33]. MARS can be thought of as piecewise regression in which the data are fit using a defined number of linear splines. This method is particularly useful if a species’ response to the environment is presumed to be non-monotonic [34].

Machine learning (ML) approaches, including classification methods, are ‘data-driven’. Unlike statistical approaches, which assume an initial data model (e.g., linear or logistic response), and specific distribution functions, ML attempts to learn the relationship by finding dominant patterns in the data [35]. Classification methods use a series of branching decisions regarding the predictor variables to partition the response variable into homogeneous groups (terminal nodes or leaves). These decision trees can then be used as a stand-alone classifier or in conjunction with other methods to build more robust predictions. One such approach to develop more robust classifiers is a method called random forests [35]. This approach uses a collection of classification trees, each trained on a random subset of the data. Each tree uses the predictor variables from a given data point to ‘vote’ for the most popular class for that input [35].

Boosted regression trees (BRTs) bridge the traditional statistical techniques such as GLMs and ML. Instead of relying on a ‘popular vote’ to determine the output class (as in RF), regression trees fit a mean response for observations within each leaf. For the logistic regression trees, the response is modeled via a logit function [36]. BRT combines the regression tree models with a method called boosting, which builds a collection of the individual trees in linear combination. As with RF and other classification, regression, or decision tree models, BRTs attempt to produce homogeneous subsets of the response variable by partitioning the multivariate predictor space [36].

Maximum entropy (MaxEnt) modeling also began as a general-purpose ML tool to make predictions from incomplete information [37]. This method attempts to estimate a target probability distribution of occurrence conditional on environmental variables by finding the distribution of maximum entropy subject to constraints defined by the target sample [37]. These constraints consist of five feature types that reflect the relationship between the probability distribution and environmental variables: linear, quadratic, product, threshold, and binary. It has since been shown to be mathematically equivalent to logistic regression under certain circumstances [10,37,38].

Application of these methods to species occurrence data and raster-based predictors allows us to generate high resolution estimations of geographic distributions based on environmental suitability for these species. These predictions can help increase understanding of vector distributions and target areas at risk of vector-borne pathogens. However, we have relatively little basis for understanding the differences in predicted vector distributions (and hence our projections of the human populations at risk) caused by using different modeling approaches and determining if one approach may heuristically outperform the others. In this study, we use an ensemble modeling approach to compare the performance of various models using a common set of environmental predictor data and geographically identical survey data sets of tick species occurrences. An examination of the geographic extents of suitable area conserved across models provides a starting point for evaluating the utility of these different modeling approaches.

## 2. Materials and Methods

### 2.1. Tick Distribution Data

We used georeferenced records from three years of tick collections performed from late 2015 through 2018 containing observations of adult *Amblyomma americanum*, *Dermacentor variabilis*, or *Ixodes scapularis* [39,40]. The initial dataset included 1956 surveys of presence or absence for each of the three species. Spatially, the dataset contained multiple transects spread across 41 sites in mainland Florida. Within each site, pairs of transects were run in the primary local biotopes (2–10 transects, depending on the size and heterogeneity of land cover at each site). In aggregate, sampling of the major biotopes was performed proportional to the state-wide land class coverages. Temporally, these transects were repeated regularly throughout the study period (repeated surveys of each transect). Collection efforts occurred year-round to ensure all sites were sampled with the same intensity regardless of when individual species’ peak seasonal activity occurred [39,40]. The methods for collection are described in further detail elsewhere [39,40]. The repeated sampling of multiple biotopes within sites produced a total of 1956 presence or absence records for the dataset.

As many of the environmental data layers were in raster format, a one-hectare vector grid matching the extent and resolution of the environmental predictors was generated. The geographic coordinates for the midpoints of the 1956 survey records was overlain on the vector grid. All surveys within individual cells were merged to a single record and if adult ticks were found during any of those surveys, that species was considered ‘present’ in that cell, otherwise it was declared absent. Thus, ‘absence’ cells never yielded adult ticks for an individual species throughout the entire study, while grid cells containing one or more occurrence records were considered ‘presence’ locations. In some cases, the same transect was linked to multiple grid cells during different surveys, primarily due to slight modifications in the position of the transect as a result of changes in the local environment such as prolonged flooding, burning, and other factors impacting accessibility of a transect location. In these cases, records were aggregated in the grid cell within which they occurred, rather than to the grid cell containing previous records of the same transect. The coordinates for the geometric centers of presence or absence cells was extracted and used to delineate the location of each record, resulting in a dataset of 560 points for subsequent modeling (Table 1).

### 2.2. Selection of Environmental Predictors

Environmental predictors used in our species distribution models consisted of 36 characteristics describing the climatic and habitat conditions in the study area. Habitat variables included three descriptions of Normalized Difference Vegetation Index (NDVI; minimum, mean, and maximum), land cover, elevation, soil and geomorphologic characteristics, and distance from water features. The climatic characteristics considered were 19 measures of temperature and precipitation variability.

Variables describing NDVI, elevation, soil, and geomorphologic characteristics were derived from MODIS NDVI 16-day composites, ASTER Global DEM, STATSGO soils database and national hydrography datasets, respectively [41,42,43,44]. The land cover classification was derived from the Florida Cooperative Land Cover database, which includes statewide classifications of all major land cover types at a native resolution of 10 m [45]. The database represents a hierarchical classification. The primary state-level land cover types were aggregated using a majority rule to the coarsest level as one of five primary types: Forest, which included pine and hardwoods; shrub, which encompassed shrub and brush lands; grasslands; wetlands; and a final, general category including all other land types such as water bodies, urban areas, and seasonal agriculture. All environmental predictors were resampled using bilinear interpolation for continuous variables and majority rule for categorical variables and cropped to the same extent and 1 ha resolution.

The climatic variables consisted of 19 bioclimatic variables calculated from gridded daily temperature and precipitation estimates from Daymet [46]. The climatic variables follow the same naming conventions and are calculated following the methodology of Hijmans et al. (2005) as implemented in the ‘biovars’ function in the dismo R package [47,48]. The bioclimatic variables were calculated at the native 1 km^2^ resolution of the gridded daily estimates.

### 2.3. Distribution Models

Initial variable screening was performed before construction and selection of our distribution models to reduce the potential for collinearity. Reduction of the potential variable set was guided by the procedures outlined in Springer et al. (2015). First, Pearson, Spearman, and Kendall correlation coefficients were calculated amongst all environmental predictors. Univariate GAMs were constructed to assess initial significant relationships between each predictor and the dependent variable. Predictors were ranked by the deviance explained of the dependent variable. Deviance explained is a GAM parameter analogous to R-squared used GLMs and represents the amount of variation in the dependent variable that is explained by the given predictor [11]. To produce the final set of variables for consideration in the distribution models, the variable with the highest deviance explained from the univariate GAM was selected for inclusion. All additional variables were selected in descending order by deviance explained and whether all pairwise correlations with previously selected variables was below a ±0.7 threshold [49].

We used five different modeling algorithms: (1) general linear models (logistic regression—LR) [33], (2) boosted regression trees (BRTs) [36], (3) random forests (RF) [35], (4) multivariate adaptive regression splines (MARS) [34], and (5) maximum entropy (MaxEnt) [37], to estimate the distribution of each species. Each of these methods are generally considered to perform well using presence/absence or presence/background data [4,11]. It should be noted that MaxEnt is generally run as a ‘presence-only’ model, with background data drawn randomly from the study area, although it can be supplied with spatially biased pseudo-absences [50,51]. Here, MaxEnt was supplied with the same (‘true’) absence data as the other algorithms in place of the random background draw.

Each model algorithm was run through an iterative procedure to select the best model from the set of environmental predictors and was optimized using performance metrics specific to each method. The optimized GLM was selected by performing an exhaustive search of first order interactions in the model space. Competing models were compared using the small sample size corrected Akaike’s Information Criterion (AICc) and the final model was selected by minimizing this value [52,53]. The BRT model was optimized by varying tree complexity, learning rate, and ‘bag fraction’ to select a model minimizing mean deviance with at least 1000 trees according to Elith et al. (2008). The random forest model was optimized by varying the number of considered variables, node size, and sample size to minimize out-of-bag (OOB) error [36]. The MARS model selection procedure utilized an internal 10-fold cross validation procedure to select the appropriate variables and number of knots [34,54]. As with the GLM model, we only considered first-order interactions in the MARS model. The optimized model was selected to maximize the GRSq, which is a cross validated estimate of the predictive power of the model [54]. The MaxEnt model was selected by varying the betamultiplier with a contribution threshold of five percent and maximum correlation threshold between variables of 0.9. Optimization of these parameters allows for a certain amount of uncertainty to be introduced into the model and limits the inclusion of extraneous variables (low contribution) and collinearity. The optimal model was selected to maximize the Area Under the Curve (AUC) of internally withheld test data [55].

Validation of the final models was performed using 10-fold cross validation to assess the agreement between observed and predicted values. The dataset used to select the optimized model was divided into 10 folds. Each model was trained by withholding one fold of the data and running the model with the remaining folds. The withheld fold was used to test the predictive accuracy of each run. This procedure was repeated ten times so that each fold was withheld and used for testing. All models were validated using the same folds for consistency across methods.

### 2.4. Spatial Predictions

Estimated habitat suitability for each species was determined by applying the optimized algorithms to a set of raster variables. The output is a probability surface for the entire mainland portion of the state, representing the probability that the conditions in a given raster cell are suitable for each species. These probability surfaces were reclassified as suitable or unsuitable based on a specified threshold criterion for prediction accuracy (sensitivity = specificity). This threshold criterion was selected as a tradeoff between predicting true positives and true negatives. The ensemble prediction of the distribution of suitable habitat for each species is the agreement between models and was assessed by summing the binary suitability estimates for each method. Thus, suitability was based on a consensus score ranging from 0–5 ranking how many of the algorithms indicated a given location was suitable or not. We also retained the continuous probability estimates for each optimized model to visualize single model variation from the ensemble prediction.

## 3. Results

### 3.1. Predictor Variables

The correlation matrices and the GAM variable screening procedure reduced the set of 36 potential environmental covariates to 17 variables for *A. americanum*, 17 for *I. scapularis*, and 18 for *D. variabilis*. Several variables were important in models for all species, although there was some variation in the deviance they explained and their rank (Table 2). Variables capturing the extremes in climate patterns were frequently included for consideration, as were one or more measures of vegetation health or greenness. Average temperatures during the driest quarter, total precipitation, and precipitation during the wettest and driest months were considered for all three species. Mean NDVI was also considered for all species. Elevation, distance to water bodies and curvature, a geomorphological characteristic describing low-lying areas where water may pool transiently, were also considered for all species. Consideration of these variables are consistent with previous studies of tick distributions, which have selected variables either a priori or through similar selection procedures [7,8,9,10,11].

The final variables included in each model are listed in Table 3. Selection of final models for each species indicated that several variables were conserved across algorithms. Most frequently, annual precipitation (Bio12), mean temperature of the driest quarter (Bio9), minimum temperature of the coldest month (Bio6), and mean NDVI were included in the final models for each species. The conservation of these variables across algorithms and species is indicative of the similar ecological niches occupied by these ticks.

All the modeling algorithms performed comparably well for a given species. The cut off threshold for delineating presence from absence ranged from 0.06–0.3 which is on par with the thresholds for delineating presence used by James et al. (2015) and Kessler et al. (2018). The cross-validated AUC scores for *A. americanum* were all between 0.89 and 0.92. The AUC scores for *I. scapularis* and *D. variabilis* were slightly lower, with ranges of 0.83–0.90 and 0.76–0.83, respectively (Table 4). We found that all the algorithms displayed reduced performance when the imbalance between response classes (presence vs. absence) increased, which is likely a result of the smaller ‘presence’ sample sizes for *I. scapularis* and *D. variabilis*. For each of the species BRT, RF, and Logistic performed slightly better than MARS or MaxEnt.

### 3.2. Spatial Predictions

**Figure 1:**
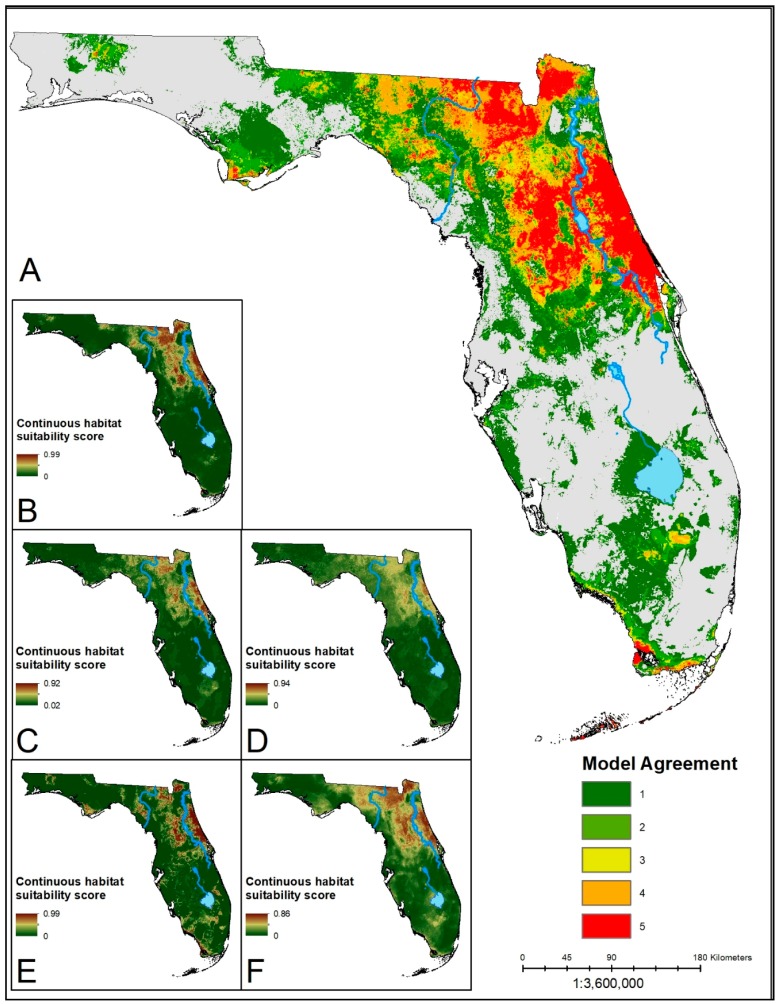
(**A**) Ensemble prediction of suitable habitat for *A. americanum*. Hotter colors indicate higher agreement in the number of models predicting suitability of an area. (**B**–**F**) Continuous suitability scores for the five modeling algorithms: LR, BRT, RF, MARS, MaxEnt, respectively. A core region in the north-central region of the state shows consensus across all five algorithms. Suitable areas in the southern part of the state are sparser, with lower model agreement.

**Figure 2:**
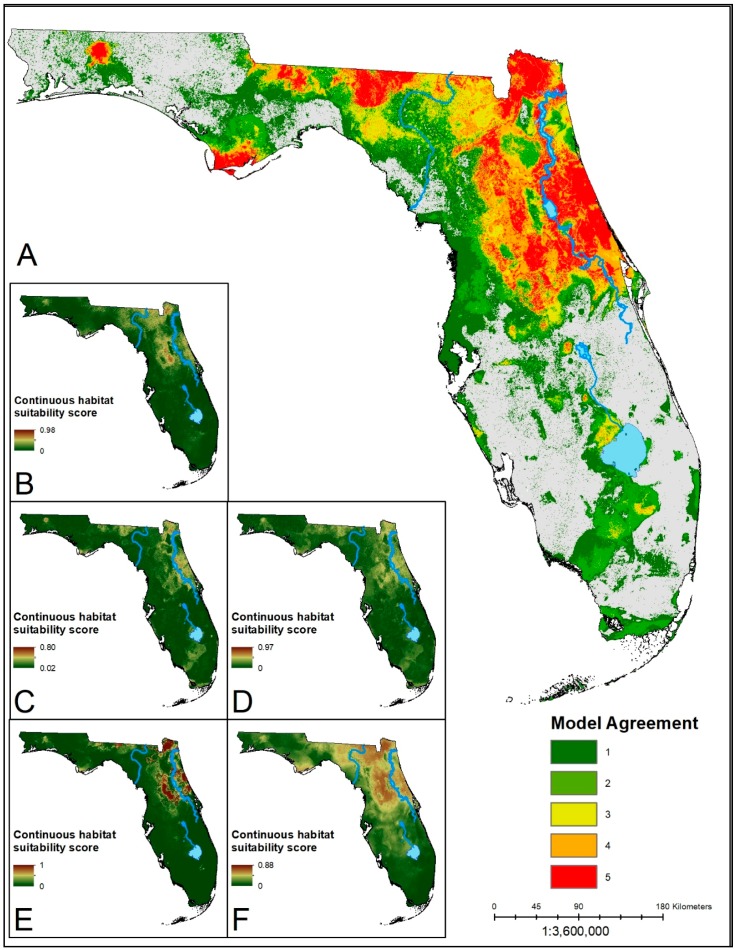
(**A**) Ensemble prediction of suitable habitat for *I. scapularis*. Hotter colors indicate higher agreement on habitat suitability across models for a given area. (**B**–**F**) Continuous suitability scores for the five modeling algorithms: LR, BRT, RF, MARS, MaxEnt, respectively. Much of the northeastern part of the state is deemed suitable by the majority of the models. Southern areas of predicted suitability show lower consensus.

**Figure 3:**
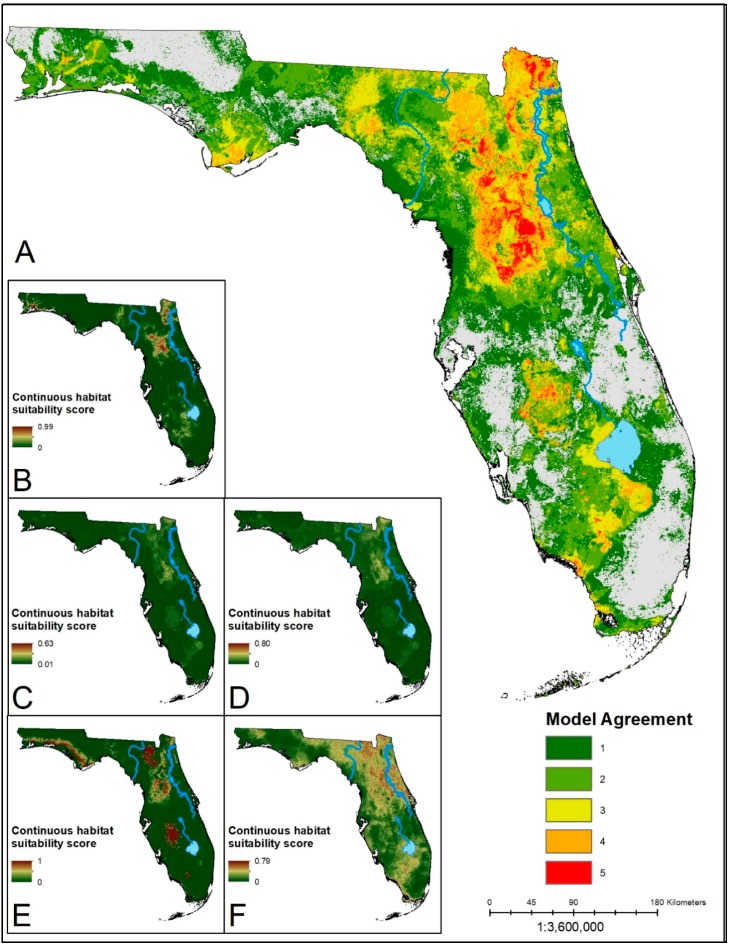
(**A**) Ensemble prediction of suitable habitat for *D. variabilis*. Hotter colors indicate higher agreement in habitat suitability. (**B**–**F**) Continuous suitability scores for the five modeling algorithms: LR, BRT, RF, MARS, MaxEnt, respectively. Overall there is lower consensus on suitable habitat across the state. However, there is greater model agreement in the southern part of the state for this species than for black-legged or lone star ticks.

The distributions of suitable habitat produced from the ensemble predictions for each species showed similar corridors of high model agreement through the center of the Florida peninsula (Figure 1, Figure 2 and Figure 3). Each ensemble map identified a contiguous area of suitability in North Central Florida, with generally reduced suitability in the southern portions of the state. In the South Central region of the state, the Lake Whales Ridge is identifiable in ensemble predictions for *Amblyomma americanum* and *Ixodes scapularis* as either unsuitable or suitable, respectively. The southwestern coast, however, from Naples south into Everglades National Park shows an area of predicted suitability that is conserved across models and species. The panhandle of the state is similarly less suitable for each of the species except for a disjunct region in the heavily forested northwestern corner of the state. The ensemble predictions for *A. americanum* and *I. scapularis* show high consensus across algorithms with more area deemed suitable by three or more algorithms. The prediction for *D. variabilis* showed greater discordance, with a greater area having only one or two algorithms agreeing on suitability; lower agreement for this species may be a result of the small sample size used for building the models. For all species, the MARS algorithms predicted the least amount of area as suitable habitat, and with the highest mean suitability scores. This could be a sign of overfitting in the model.

## 4. Discussion

The estimated distributions for the medically important tick species presented in this study show strikingly similar patterns. Much of northern Florida is considered suitable by the majority of—or all of—the modeling algorithms used, while suitability decreases at lower latitudes. The deviance explained, which is a measure of how well the model explains the relationship between dependent and independent variables, by any single predictor was very low for all species, with the greatest deviance explained being mean temperature of the coldest quarter (0.253), mean temperature of the wettest quarter (0.20), and mean diurnal range (0.137) for *A. americanum*, *I. scapularis*, and *D. variabilis*, respectively. This is indicative that a plethora of conditions influence suitability for these species and distributions are not driven by a predominant single factor. We found that while consensus among algorithms was high (as indicated by large regions of suitable habitat receiving a score of ≥3 in the consensus projections), each algorithm produced some variation in predicted suitable area. The use of an ensemble approach provides a degree of certainty that the estimated distribution reflects that of the species and is not simply a result of the choice of modeling algorithm.

However, it is important to remember that the consensus score of ensemble predictions are simple sums, so they could be disproportionately influenced by conservative SDM algorithms [5]. For example, in a study of invasive fish species in the United States, Marmion et al. (2009) found that MaxEnt produced estimated distributions with the smallest geographic area that did not extend much beyond existing presence records (although the model’s training AUC values were the highest of all models in their ensembles). As a result, the ensemble maps largely reflected the distributional estimates from their MaxEnt models. In our study, AUC values for each of the three species were quite close, indicating that the predictive performance of each algorithm was similar. However, MaxEnt consistently produced the lowest AUC values while producing the predictions of suitable area with the largest geographic extents for each species. This produces much of the low model agreement areas (green) in our predictions. Conversely, the MARS algorithm produced higher AUC values but was most conservative in its estimations of suitable area, which highlights one of the main criticisms of ensemble approaches; while MARS and other algorithms predict suitable conditions in the same areas, the extent of the MARS predictions limits the areas where highest model agreement is possible. Ultimately, the accuracy of the various models for these tick distributions in Florida will need to be determined by validation surveys in previously unsampled regions of the state. These studies are currently underway and will be completed following the annual activity periods of the adult ticks for each species.

In this study we limited our observations to occurrence records of adult specimens collected via flagging, which resembles casual human exposure more than other sampling techniques such as chemical attractants/traps or collections from sentinel species. Collections from free-ranging wildlife may be a more sensitive representation of tick burden on the landscape but make high resolution spatial and temporal predictions difficult because hosts may range widely through multiple habitat types, and ticks may be attached to hosts for multiple days [56]. Additionally, some tick species that have preferences for wildlife species rarely attach to humans. A primary utility of these records, however, involved validating (or refuting) the SDMs. Previous publications using other survey methods, including collections from wildlife, indicated the occurrence of lone star, black-legged, and American dog ticks across northern Florida, which supports the distributional estimates presented here [57,58,59]. However, records in the literature also note the presence of these species from regions in south Florida, although these historic records are sparse [57,60]. Although we rarely found questing ticks in southern Florida, several of the SDMs indicated suitable conditions broadly throughout the region. These regions might represent some of the best evaluations of alternative SDMs within the ensemble suite as they provide the clearest distinction in predictions. Sites where the five models either all predicted occurrence or absence provide little discriminating power concerning which modeling approach might be “best”. It is unclear if the north–south trend in suitability represents a true lack of suitable habitat in this region [61,62]. The meridional trend in *A. americanum* abundance [57] closely corresponds to the three primary ecoregions in Florida, implicating variable ecological conditions in the observed distributional patterns. Conversely, the predicted occurrences of tick species further south for some of the models, especially the general agreement among models in the furthest southwestern regions of the state, suggest there may be local foci of infestations that deserve further evaluation.

Previous studies modeling the distributions of these species across the United States and North America have captured similar general trends, although the resolution for these studies is spatially and temporally coarser (resolved to the county level for all available times). Our observed patterns in distribution for *A. americanum* are supported by the county-level analysis of the contiguous United States generated by Springer et al. [61], which observed reduced agreement for the suitability of southern (and southeastern especially) Florida. Hahn et al. (2016) observed reduced model agreement for south Florida in predictions for *I. scapularis* suitability. Similarly, the predicted distribution of suitable habitat for *D. variabilis* was supported by previous studies showing much of the state as suitable for the species, although the southeastern regions are less so [9,10]. The distribution estimate by James et al. (2015) additionally captured the area of higher suitability along the gulf coast of the panhandle for *D. variabilis*.

A practical application of high resolution ensemble models involves case investigations in regions where tick-borne pathogens are or might be emerging. Reported cases of locally acquired tick-borne infections in Florida are relatively rare compared to numbers in the Northeast and Upper mid-western United States. Additionally, they tend to be geographically scattered, making investigations challenging and expensive. The disparate occurrence of cases may reflect variation in the types of human activities that influence risk and/or variation in the distribution of vector species or pathogen. The ensemble models, if validated, could allow local and state health officials to target surveillance and other intervention strategies while minimizing marginal costs of staff, equipment and supplies.

Historic under-sampling of ticks in many counties in Florida likely produces poorly characterized distributions and estimates of human risk of exposure. The spatial predictions of questing Ixodid ticks in Florida produced by this research contributes to a better understanding of exposure risk to ticks and their pathogens by providing an initial indication of risky landscapes and may inform control measures by county, state, and federal agencies. Understanding the distributions for pathogen vectors, at high spatial resolutions, can be an important first step to identifying and targeting areas at risk for vector-borne diseases. If the maps are determined to be spatially accurate and temporally stable, they could also help diagnostically by ruling out certain species-associated pathogens based on geographic activity of the patient. This is evident in the consistently high negative predictive values for ruling out species distributions. The heterogeneous distributions of the three tick species covered by this study should help guide future work on evaluating human exposure risk to these vectors and their pathogens in Florida. We found numerous temperature and precipitation variables were important in predicting the distribution of adult lone star, black-legged, and American dog ticks in the state and the variables were conserved across model algorithms and species. Additional models informed by tick densities, pathogen prevalence, or disease incidence may better improve estimates of exposure risk to tick-borne diseases, as these approaches address important aspects of transmission not addressed by habitat suitability alone [63,64].

## 5. Conclusions

Broad patterns of environmental suitability for the three species modeled are similar. This finding speaks to the similarity in niches occupied by these species. The ecology of all three species is largely driven by similar responses to climatic conditions such as temperature and moisture availability. The northern and north central regions of the state consist of a large contiguous tract of suitable area that diminishes at southern latitudes.

The fine-grained distributions of suitable habitat for medically-important tick species in Florida are more heterogeneous than previous studies would suggest. Individually, each modeling algorithm produced estimates with closely similar accuracies and geographic distributions of suitability. By using an ensemble of multiple algorithms, we developed consensus predictions which highlighted suitable areas conserved across models. The high degree of consensus among algorithm predictions suggests that although each approach produces some noise, no single algorithm vastly outperformed the others. Each algorithm’s prediction should be considered individually, however, as high accuracy measures could be due to overfitting and produce low generalizability. As a result, reliance on any single algorithm carries a degree of uncertainty in its prediction. Validation surveys are required to test these predictions. When used in conjunction with additional behavioral or exposure factors to estimate risk of exposure to ticks or tick-borne pathogens, fine resolution habitat suitability models may be a useful tool for identifying areas of tick presence.

## Figures and Tables

**Table 1:** Number of presence/absence observations used for modeling each species. The total number (*n* = 560) and geographic location of observations is conserved for each species, with only observed occurrence (presence) varying.

Species	Total	Presence	Absence
*Amblyomma americanum*	560	98	462
*Ixodes scapularis*	560	65	495
*Dermacentor variabilis*	560	30	530

**Table 2:** Deviance explained by considered variables and rank via univariate generalized additive models (GAM) for each species. NDVI is the Normalized Difference Vegetation Index, a proxy for vegetation health.

Variable	*Amblyomma americanum*		*Ixodes scapularis*		*Dermacentor variabilis*	
	Rank	Deviance Explained	Rank	Deviance Explained	Rank	Deviance Explained
Mean Diurnal Range (Bio2)	----	----	----	----	1	0.137
Isothermality (Bio3)	19	0.078	----	----	23	0.006
Tmax of Warmest Month (Bio5)	----	----	----	----	----	----
Tmin of Coldest Month (Bio6)	----	----	3	0.16	----	----
Tmean of Wettest Quarter (Bio8)	----	----	1	0.201	14	0.025
Tmean of Driest Quarter (Bio9)	8	0.169	11	0.102	6	0.096
Tmean of Coldest Quarter (Bio11)	1	0.253	----	----	----	----
Annual Precipitation (Bio12)	6	0.226	2	0.163	8	0.067
Precipitation of Wettest Month (Bio13)	5	0.232	9	0.106	10	0.047
Precipitation of Driest Month (Bio14)	13	0.151	21	0.026	22	0.007
Precipitation Seasonality (Bio15)	14	0.15	----	----	9	0.054
Precipitation of Driest Quarter (Bio17)	----	----	10	0.103	----	----
Curvature (curv)	23	0.027	25	0.004	26	0.0004
Depth to Water (detwt)	----	----	23	0.011	16	0.013
Distance to Water (distwater)	24	0.026	24	0.007	11	0.039
Elevation (DEM)	26	0.012	26	0.003	21	0.01
Maximum NDVI (NDVImax)	----	----	----	----	15	0.02
Mean NDVI (NDVImean)	10	0.167	16	0.059	20	0.01
Minimum NDVI (NDVImin)	----	----	18	0.044	24	0.005

Tmax = Maximum Temperature in Celsius; Tmean = Mean Temperature in Celsius; Tmin = Minimum Temperature in Celsius; NDVI = Normalized Difference Vegetation Index.

**Table 3:** Variables included in final models for each species. The machine learning algorithms consider all variables initially, and unimportant variables are pruned back. As a result, all considered variables are listed for these models, though the contribution of some variables is quite small.

Species	Final Model Variables
*Amblyomma americanum*	
LR	Bio13 + Bio12 + Bio9 + NDVImean + Bio3 + curv + distwater + dem + shrub
BRT	Bio12 + NDVImean + Bio13 + Bio9 + Bio14 + Bio15 + distwater + Bio3 + curv + dem + NDVImin + Bio11 + detwt + forest + wetlands + shrub + grass
RF	Bio11 + Bio13 + Bio12 + Bio9 + NDVImean + Bio14 + Bio15 + dtwt + Bio3 + curv + distwater + NDVImin + DEM + forest + grass + shrub + wetlands
MARS	Bio12 + NDVImean + Bio14 + Bio13 + distwater + DEM + Bio3 + NDVImin
MaxEnt	Bio12 + Bio9 + NDVImean + distwater
*Ixodes scapularis*	
LR	Bio12 + Bio6 + distwater + curv + shrub + wetlands
BRT	Bio12 + Bio13 + Bio17 + Bio8 + NDVImean + Bio9 + NDVImin + distwater + Curv + Bio14 + DEM + Bio6 + detwt + wetlands + shrub + forest + grass
RF	Bio8 + Bio12 + Bio6 + Bio13 + Bio17 + Bio9 + NDVImean + NDVImin + Bio14 + detwt + distwater + curv + DEM + forest + grass + shrub + wetlands
MARS	Bio12 + Bio6 + Bio8 + Bio13 + curv
MaxEnt	Bio12 + Shrub + Bio6 + distwater + Bio13
*Dermacentor variabilis*	
LR	Bio2 + Bio9 + Bio15 + Bio13 + NDVImax + DEM + Bio14 + curv
BRT	Bio12 + Bio15 + Bio9 + NDVImax + Bio3 + Bio13 + Bio2 + distwater + NDVImin + Bio14 + DEM + curv + Bio8 + forest + detwt + shrub + grass + wetlands
RF	Bio2 + Bio9 + Bio12 + Bio15 + Bio13 + distwater + Bio8 + NDVImax + detwt + DEM + Bio14 + Bio3 + NDVImin + curv + forest + grass + shrub + wetlands
MARS	Bio2 + Bio13 + Bio15 + Bio3 + Bio15 + Bio3 + detwt + NDVImax + curv + NDVImin
MaxEnt	Bio12 + NDVImax + forest + grass

LR = Logistic Regression; BRT = Boosted Regression Trees; RF = Random Forests; MARS = Multivariate Adaptive Regression Splines; MaxEnt = Maximum Entropy.

**Table 4:** Performance metrics associated with each of the five modeling algorithms for each species. AUC ranges correspond to the upper and lower bounds of a 95% CI calculated from across the 10-fold cross validation.

	Threshold ^1^	AUC ^2^ (95% C.I.)	s.e. ^3^	Accuracy ^4^ (95% C.I.)	Kappa ^5^	Sensitivity ^6^	Specificity ^7^	Positive Predictive Value ^8^	Negative Predictive Value ^9^
*Amblyomma americanum*									
Logistic	0.18	0.90 (0.87–0.93)	0.0152	0.828 (0.794–0.859)	0.525	0.827	0.829	0.506	0.957
BRT	0.26	0.92 (0.89–0.95)	0.0143	0.934 (0.91–0.953)	0.792	0.939	0.933	0.748	0.986
RF	0.23	0.92 (0.89–0.95)	0.0151	0.916 (0.89–0.938)	0.749	0.959	0.907	0.686	0.991
MARS	0.3	0.92 (0.89–0.94)	0.0132	0.889 (0.86–0.914)	0.660	0.847	0.898	0.638	0.965
MaxEnt	0.13	0.89 (0.85–0.92)	0.0168	0.617 (0.575–0.658)	0.276	0.959	0.544	0.309	0.984
*Ixodes scapularis*									
Logistic	0.14	0.83 (0.78–0.88)	0.0254	0.923 (0.898–0.944)	0.693	0.923	0.923	0.612	0.989
BRT	0.16	0.88 (0.83–0.92)	0.0227	0.794 (0.758–0.827)	0.367	0.785	0.796	0.336	0.966
RF	0.09	0.90 (0.86–0.93)	0.0188	0.878 (0.848–0.904)	0.593	1.000	0.862	0.489	1.000
MARS	0.15	0.84 (0.78–0.89)	0.0279	0.875 (0.844–0.901)	0.524	0.785	0.887	0.477	0.969
MaxEnt	0.24	0.85 (0.80–0.90)	0.0241	0.556 (0.514–0.598)	0.166	0.923	0.508	0.198	0.980
*Dermacentor variabilis*									
Logistic	0.06	0.82 (0.77–0.87)	0.0246	0.809 (0.773–0.84)	0.244	0.800	0.809	0.192	0.986
BRT	0.1	0.82 (0.76–0.88)	0.0301	0.952 (0.931–0.968)	0.659	0.967	0.951	0.527	0.998
RF	0.04	0.83 (0.78–0.88)	0.0265	0.86 (0.829–0.888)	0.383	1.000	0.853	0.278	1.000
MARS	0.14	0.77 (0.69–0.86)	0.0424	0.916 (0.89–0.938)	0.443	0.733	0.926	0.361	0.984
MaxEnt	0.14	0.76 (0.69–0.83)	0.0355	0.358 (0.318–0.399)	0.044	0.967	0.323	0.075	0.994

^1^ Continuous probability score used to delineate presence from absence for consensus predictions (sensitivity = specificity); ^2^ area under the Receiver Operating Characteristics (ROC) curve; ^3^ standard error of AUC; ^4^ ratio of sum of correctly predicted positives and negatives to the sample size; ^5^ Kohen’s Kappa, a measure of agreement that accounts for agreement due to chance; ^6^ true positive rate; ^7^ true negative rate; ^8^ proportion of positives that are true positives; ^9^ proportion of negatives that are true negatives.

## References

[1] Health F.D.O. (2017). Reportable Diseases Frequency Report.

[2] Guisan A., Zimmermann N.E. (2000). Predictive habitat distribution models in ecology. Ecol. Model..

[3] Blackburn J.K., O’Connell K.P., Skowronski E.W., Sulakvelidze A. (2010). Integrating geographic information systems and ecological niche modeling into disease ecology: A case study of *bacillus anthracis* in the United States and Mexico. Emerging and Endemic Pathogens: Advances in Surveillance, Detection, and Identificiation.

[4] Elith J., Graham C.H. (2009). Do they? How do they? Why do they differ? On finding reasons for differing performances of species distribution models. Ecography.

[5] Marmion M., Parviainen M., Luoto M., Heikkinen R.K., Thuiller W. (2009). Evaluation of consensus methods in predictive species distribution modelling. Divers. Distrib..

[6] Araujo M.B., New M. (2007). Ensemble forecasting of species distributions. Trends Ecol. Evol..

[7] Brownstein J.S., Holford T.R., Fish D. (2003). A climate-based model predicts the spatial distribution of the lyme disease vector *ixodes scapularis* in the United States. Environ. Health Perspect..

[8] Hahn M.B., Jarnevich C.S., Monaghan A.J., Eisen R.J. (2016). Modeling the geographic distribution of ixodes scapularis and ixodes pacificus (acari: Ixodidae) in the contiguous United States. J. Med. Entomol.

[9] James A.M., Burdett C., McCool M.J., Fox A., Riggs P. (2015). The geographic distribution and ecological preferences of the american dog tick, dermacentor variabilis (say), in the USA. Med. Vet. Entomol.

[10] Minigan J.N., Hager H.A., Peregrine A.S., Newman J.A. (2018). Current and potential future distribution of the american dog tick (dermacentor variabilis, say) in North America. Ticks Tick Borne Dis..

[11] Springer Y.P., Jarnevich C.S., Barnett D.T., Monaghan A.J., Eisen R.J. (2015). Modeling the present and future geographic distribution of the lone star tick, amblyomma americanum (ixodida: Ixodidae), in the continental United States. Am. J. Trop. Med. Hyg..

[12] Beard C.B.E.R.J., Barker C.M., Garofalo J.F., Hahn M., Hayden M., Monaghan A.J., Ogden N.H., Schramm P.J. (2016). The Impacts of Climate Change on Human Health in the United States: A Scientific Assessment.

[13] Telford S.R., Dawson J.E., Katavolos P., Warner C.K., Kolbert C.P., Persing D.H. (1996). Perpetuation of the agent of human granulocytic ehrlichiosis in a deer tick-rodent cycle. Proc. Natl. Acad. Sci. USA.

[14] Spielman A. (1976). Human babesiosis on nantucket island: Transmission by nymphal ixodes ticks. Am. J. Trop. Med. Hyg..

[15] Johnson D.K., Schiffman E.K., Davis J.P., Neitzel D.F., Sloan L.M., Nicholson W.L., Fritsche T.R., Steward C.R., Ray J.A., Miller T.K. (2015). Human infection with ehrlichia muris-like pathogen, United States, 2007–2013. Emerg. Infect. Dis..

[16] Pritt B.S., Sloan L.M., Johnson D.K., Munderloh U.G., Paskewitz S.M., McElroy K.M., McFadden J.D., Binnicker M.J., Neitzel D.F., Liu G. (2011). Emergence of a new pathogenic ehrlichia species, wisconsin and minnesota, 2009. N. Engl. J. Med..

[17] Lane R.S., Piesman J., Burgdorfer W. (1991). Lyme borreliosis: Relation of its causative agent to its vectors and hosts in North American and Europe. Annu. Rev. Entomol..

[18] Hopla C.E. (1953). Experimental studies on tick transmission of tularemia organisms. Am. J. Hyg..

[19] Hopla C.E. (1955). The multiplication of tularemia organisms in the lone star tick. Am. J. Hyg..

[20] Anderson B.E., Greene C.E., Jones D.C., Dawson J.E. (1992). Ehrlichia ewingii sp. Nov., the etiologic agent of canine granulocytic ehrlichiosis. Int. J. Syst. Bacteriol..

[21] Anderson B.E., Sims K.G., Olson J.G., Childs J.E., Piesman J.F., Happ C.M., Maupin G.O., Johnson B.J. (1993). Amblyomma americanum: A potential vector of human ehrlichiosis. Am. J. Trop. Med. Hyg..

[22] Ledin K.E., Zeidner N.S., Ribeiro J.M., Biggerstaff B.J., Dolan M.C., Dietrich G., Vredevoe L., Piesman J. (2005). Borreliacidal activity of saliva of the tick amblyomma americanum. Med. Vet. Entomol..

[23] Masters E.J., Grigery C.N., Masters R.W. (2008). Stari, or masters disease: Lone star tick-vectored lyme-like illness. Infect. Dis. Clin. N. Am..

[24] Goddard J., Varela-Stokes A.S. (2009). Role of the lone star tick, amblyomma americanum (L.), in human and animal diseases. Vet. Parasitol..

[25] Childs J.E., Paddock C.D. (2003). The ascendancy of amblyomma americanum as a vector of pathogens affecting humans in the United States. Annu. Rev. Entomol..

[26] Felz M.W., Durden L.A., Oliver J.H. (1996). Ticks parasitizing humans in Georgia and South Carolina. J. Parasitol..

[27] Armstrong P.M., Brunet L.R., Spielman A., Telford S.R. (2001). Risk of lyme disease: Perceptions of residents of a lone star tick-infested community. Bull. World Health Organ..

[28] Philip C.B., Jellison W.L. (1934). The american dog tick, dermacentor variabilis, as a host of bacterium tularense. Public Health Rep..

[29] Azad A.F., Beard C.B. (1998). Rickettsial pathogens and their arthropod vectors. Emerg. Infect. Dis..

[30] Thomaes A., Kervyn T., Maes D. (2008). Applying species distribution modelling for the conservation of the threatened saproxylic stag beetle (lucanus cervus). Biol. Conserv..

[31] Stohlgren T.J., Ma P., Kumar S., Rocca M., Morisette J.T., Jarnevich C.S., Benson N. (2010). Ensemble habitat mapping of invasive plant species. Risk Anal..

[32] Miller J. (2010). Species distribution modeling. Geogr. Compass.

[33] Hosmer D.W., Lemeshow S. (1989). Applied Logistic Regression.

[34] Friedman J.H. (1991). Multivariate adaptive regression splines. Ann. Stat..

[35] Breiman L. (2001). Random forests. Mach. Learn..

[36] Elith J., Leathwick J.R., Hastie T. (2008). A working guide to boosted regression trees. J. Anim. Ecol..

[37] Phillips S.J., Anderson R.P., Schapire R.E. (2006). Maximum entropy modeling of species geographic distributions. Ecol. Model..

[38] Mount J. The Equivalence of Logistic Regression and Maximum Entropy Models. http://www.win-vector.com/dfiles/LogisticRegressionMaxEnt.pdf.

[39] Kessler W.H., Blackburn J.K., Sayler K.A., Glass G.E. (2018). Estimating the geographic distribution of host-seeking adult amblyomma americanum (acari: Ixodidae) in Florida. J. Med. Entomol..

[40] Glass G.E., Ganser C., Kessler W.H. (2019). Standardized ixodid tick surveys in mainland Florida. Insects.

[41] Spruce J.P., Gasser G.E., Hargrove W.W. (2016). Modis Ndvi Data, Smoothed and Gap-Filled, for the Conterminous Us: 2000–2015.

[42] NASA/METI/AIST/Japan Spacesystems, and U.S./Japan ASTER Science Team (2009). Aster Global Digital Elevation Model; NASA EOSDIS Land Processes DAAC. https://lpdaac.usgs.gov/products/astgtmv002/#citation.

[43] U.S. Soil Survey Staff, Natural Resources Conservation Service (2008). General Soil Map (statsgo2).

[44] Geological Survey (2004). National Hydrography Dataset.

[45] Kawula R., Redner J., Florida Fish and Wildlife Conservation (2016). Florida Cooperative Land Cover v3.2.

[46] Thornton P.E., Thornton M.M., Mayer B.W., Wei Y., Devarakonda R., Vose R.S., Cook R.B. (2018). Daymet: Daily Surface Weather Data on a 1-km Grid for North America.

[47] Hijmans R.J., Cameron S.E., Parra J.L., Jones P.G., Jarvis A. (2005). Very high resolution interpolated climate surfaces for global land areas. Int. J. Climatol..

[48] Hijmans R.J., Phillips S.J., Leathwick J.R., Elith J. (2017). Package Dismo-Species Distribution Modeling (v 1.0-12). Circles.

[49] Dormann C.F., Elith J., Bacher S., Buchmann C., Carl G., Carré G., Marquéz J.R.G., Gruber B., Lafourcade B., Leitão P.J. (2013). Collinearity: A review of methods to deal with it and a simulation study evaluating their performance. Ecography.

[50] Guillera-Arroita G., Lahoz-Monfort J.J., Elith J., O’Hara R.B. (2014). Maxent is not a presence-absence method: A comment on thibaud et al. Methods Ecol. Evol..

[51] Phillips S.J., Dudik M., Elith J., Graham C.H., Lehmann A., Leathwick J., Ferrier S. (2009). Sample selection bias and presence-only distribution models: Implications for background and pseudo-absence data. Ecol. Appl..

[52] Buckland S.T., Burnham K.P., Augustin N.H. (1997). Model selection: An integral part of inference. Biometrics.

[53] Calcagno V., de Mazancourt C. (2010). Glmulti: An r package for easy automated model selection with (generalized) linear models. J. Stat. Softw..

[54] Milborrow S., Hastie T., Tibshirani R. (2011). Derived from mda:mars by T. Hastie and R. Tibshirani. Earth: Multivariate adaptive regression splines. R Package. http://www.milbo.org/doc/earth-notes.pdf.

[55] Jueterbock A., Smolina I., Coyer J.A., Hoarau G. (2016). The fate of the arctic seaweed fucus distichus under climate change: An ecological niche modeling approach. Ecol. Evol.

[56] Ginsberg H.S., Ewing C.P. (1989). Comparison of flagging, walking, trapping, and collecting from hosts as sampling methods for northern deer ticks, ixodes dammini, and lone-star ticks, amblyomma americanum (acari:Ixodidae). Exp. Appl. Acarol..

[57] Allan S.A., Simmons L.A., Burridge M.J. (2001). Ixodid ticks on white-tailed deer and feral swine in Florida. J. Vector Ecol..

[58] Burroughs J.E., Thomasson J.A., Marsella R., Greiner E.C., Allan S.A. (2016). Ticks associated with domestic dogs and cats in Florida, USA. Exp. Appl. Acarol..

[59] Durden L.A., Hu R., Oliver J.H., Cilek J.E. (2000). Rodent ectoparasites from two locations in northwestern Florida. J. Vector Ecol..

[60] Durden L.A., Klompen J.S., Keirans J.E. (1993). Parasitic arthropods of sympatric opossums, cotton rats, and cotton mice from merritt island, Florida. J. Parasitol..

[61] Diuk-Wasser M.A., Gatewood A.G., Cortinas M.R., Yaremych-Hamer S., Tsao J., Kitron U., Hickling G., Brownstein J.S., Walker E., Piesman J. (2006). Spatiotemporal patterns of host-seeking ixodes scapularis nymphs (acari: Ixodidae) in the United States. J. Med. Entomol..

[62] Springer Y.P., Eisen L., Beati L., James A.M., Eisen R.J. (2014). Spatial distribution of counties in the continental United States with records of occurrence ofamblyomma americanum(ixodida: Ixodidae). J. Med. Entomol..

[63] Diuk-Wasser M.A., Hoen A.G., Cislo P., Brinkerhoff R., Hamer S.A., Rowland M., Cortinas R., Vourc’h G., Melton F., Hickling G.J. (2012). Human risk of infection with borrelia burgdorferi, the lyme disease agent, in eastern United States. Am. J. Trop. Med. Hyg..

[64] Diuk-Wasser M.A., Vourc’h G., Cislo P., Hoen A.G., Melton F., Hamer S.A., Rowland M., Cortinas R., Hickling G.J., Tsao J.I. (2010). Field and climate-based model for predicting the density of host-seeking nymphalixodes scapularis, an important vector of tick-borne disease agents in the eastern United States. Glob. Ecol. Biogeogr..

